# Factors that influence preference for male or female urologist among underserved patients in New York City

**DOI:** 10.1002/bco2.196

**Published:** 2022-10-17

**Authors:** Shirin Razdan, Patrick Ho, Christine Bieber, Michaela Sljivich, Harry Anastos, Dallin Busby, Vannita Simma‐Chiang

**Affiliations:** ^1^ Department of Urology Icahn School of Medicine at Mount Sinai Hospital New York New York USA; ^2^ St. George's University Medical School St. George's Grenada

**Keywords:** gender, office‐based, patient centred care, provider preference, urologist

## Abstract

**Objective:**

To examine the prevalence of patient preference for male or female urologic provider and explore which patient characteristics influence this preference.

**Materials and Methods:**

After obtaining hospital Institutional Review Board approval, a 14‐question survey in English and Spanish was administered across four general urology clinic sites in a single hospital system in New York City. The survey asked demographic questions and preference for a male or a female urologist. The survey included questions pertaining to the nature of the clinic visit and subsequent provider preference as well. Statistics were performed using Stata 16 (StataCorp, College Station, TX).

**Results:**

A total of 540 patients completed the 14‐question survey. The vast majority of survey respondents identified as male (90%). The largest proportion demographic groups were those aged 41–60 (47%), Hispanic or Latino (43%), Catholic (47%), unemployed (40%) and those with a high school level of education (34%). Most patients (60%) did not have a preference for a specific gender provider, whereas 37% preferred a male provider, and 3% preferred a female provider. On univariate analysis, patient age 25–40, less than high school education level and lack of employment were significant predictors of provider gender preference (*p* < 0.05), with most patients indicating a male provider preference. On multivariate analysis of gender, age, education level and employment status, gender and education level were not significant predictors of preference, whereas age 25–40 and being unemployed were significant predictors (*p* < 0.05).

**Conclusion:**

Patient gender, race and religion do not appear to influence their preference to be seen by a male or a female urologist in the clinic setting. However, patient age, unemployment and potentially educational attainment were significantly associated with a provider gender preference.

## 1 INTRODUCTION

The genitourinary history and physical exam can be anxiety‐inducing for patients, as they involve very intimate details of patient anatomy and behavioural practices.^1^ Thus, it is not uncommon to have patients request specific providers when presenting to the clinic for evaluation. This has been described extensively within the obstetrics/gynaecology literature.^2^, ^3^ Specific patient factors, including religious beliefs, education level, income and employment status, were determined to prompt a preference for a same‐gender provider.^4^, ^5^

Determining patient preference for a male or a female provider has been extensively studied across many specialties, ranging from the emergency room setting to various surgical specialties to primary care.^1^, ^6^, ^7^, ^8^ The majority of these studies have not shown a preference for a particular gender of the physician, although patient‐specific factors and more sensitive physical exams were more likely to influence preference for same‐gender physicians. Given the increased presence of female urologists in a traditionally male‐dominated field, we sought to explore what patient characteristics influenced their preference for a male or a female provider, as the latter is more likely to be encountered by patients in the years to come.

## 2 MATERIALS AND METHODS

After obtaining hospital Institutional Review Board (IRB) approval (IRB‐20‐02991), a 14‐question survey in English and Spanish was administered across four urology clinic sites in a single large hospital system in New York, NY. These clinics are general urology clinics, and appointments are usually (1) a patient's first contact with urologic care, (2) follow‐up appointments after receiving treatment or (3) preparation for surgical intervention. The patients seen at these clinics are predominantly male and frequently underserved, with many patients receiving state‐sponsored medical coverage.

A Spanish interpreter was used to create the Spanish version of the survey. The survey asked demographic questions and preference for a male or a female urologist. The survey included questions pertaining to the nature of the clinic visit and subsequent provider preference as well. (See Supporting information S1).

Clinic staff was trained to obtain informed consent and to administer the survey to patients. Surveys were given during nursing triage on arrival to the clinic. Patients had the opportunity to decline study participation without jeopardising the care they subsequently received in the clinic. Study investigators did not administer surveys to limit bias by preventing patients from seeing whether clinic urologists were male or female. This caveat to the informed consent process was discussed at length with hospital IRB who approved this specific study design given the need for this necessary blinding. Clinicians were unaware whether patients took the survey or not, as clinic staff collected surveys at the end of triage prior to escorting patients to examination rooms.

Statistics were performed using Stata 16 (StataCorp, College Station, TX). Chi‐square analysis was used to compare frequencies and to determine significant differences. Multivariable logistic regression was used to identify independent predictors of having a provider gender preference. Tests with a *p* value <0.05 were considered statistically significant.

## 3 RESULTS

From March 2021 to June 2021, 540 patients from the four urology clinic sites completed the 14‐question survey. The vast majority of survey respondents identified as male (90%), which is not unusual for the population served by these clinics. The largest proportion demographic groups were those aged 41–60 (47%), Hispanic or Latino (43%), Catholic (47%), unemployed (40%) and those with a high school level of education (34%). (Table 1).

**TABLE 1:** Patient demographics and provider preference

	Preference for provider gender		
	No (*N* = 324)	Yes (*N* = 216)	*p* value
Age (%)			
25–40 years	36 (40)	54 (60)	<0.01
41–60 years	162 (64)	90 (36)	
61–80 years	90 (63)	54 (38)	
>80 years	36 (67)	18 (33)	
Gender (%)			
Male	288 (59)	198 (41)	0.29
Female	36 (67)	18 (33)	
Race (%)			
Black or African‐American	144 (73)	54 (27)	<0.01
Hispanic or Latino	162 (69)	72 (31)	
White/Caucasian	0 (0)	72 (100)	
Other	18 (50)	18 (50)	
Religion (%)			
Christian	108 (75)	36 (25)	<0.01
Catholic	144 (57)	108 (43)	
Muslim	36 (100)	0 (0)	
Atheist	0 (0)	18 (100)	
Other	36 (40)	54 (60)	
Employment status (%)			
Employed	108 (75)	36 (25)	<0.01
Unemployed	108 (50)	108 (50)	
Retired	90 (56)	72 (44)	
Prefer not to answer	18 (100)	0 (0)	
Education (%)			
Did not complete high school	72 (64)	41 (36)	<0.01
High school or GED	90 (49)	92 (51)	
Some college, vocational or bachelor's	108 (65)	59 (35)	
Prefer not to answer	54 (69)	24 (31)	

* Chi‐squared tests used for hypothesis testing.

A total of 324 (60%) patients expressed no preference for their provider during that clinic visit. Of the 216 patients who did have a preference, 198 (92%) preferred a male provider. The only patients who had a preference for a female provider identified as female.

When stratified by demographic group, certain subgroups were significantly more likely to have a provider gender preference (Table 2). Patients aged 25–40 years were significantly more likely to have a provider gender preference (OR 2.7, 95% CI 1.6–2.4, *p* < 0.001) compared with those aged 41–60 years. The female patient's gender did not have a significant association with having a provider gender preference (OR 0.7, 95% CI 0.4–1.3, *p* = 0.29). With regard to patient race, all patients who identified as White/Caucasian (13.3% of the surveyed population) indicated a provider gender preference. However, there was no significant association of Hispanic/Latino race and provider preference compared with those who identified as Black or African‐American (OR 1.2, 95% CI 0.8–1.8, *p* = 0.43). Patients' religious affiliation was linked with provider preference. All Muslim patients (6.66% of the surveyed population) did not have a provider gender preference, whereas all identifying as atheist (3.33%) did. Christian patients were less likely to have a preference compared to the Catholic subgroup (OR 0.4, 95% CI 0.3–0.7, *p* < 0.001). Those who were unemployed or retired were significantly more likely to have a preference compared with those who were employed (OR 3.2, 95% CI 1.5–4.3, *p* < 0.001). Finally, patients who completed education beyond high school were less likely to have a preference (OR 0.5, 95% CI 0.3–0.8, *p* < 0.01).

**TABLE 2:** Univariable logistic regressions for patient preference

	OR	95% CI		*p* value
Age (years)				
41–60 (referent)	‐	‐	‐	
25–40	2.7	1.6	4.4	<0.001
61–80	1.1	0.7	1.7	0.72
>80	0.9	0.5	1.7	0.74
Female gender	0.7	0.4	1.3	0.29
Race				
Black or African‐American (referent)	‐	‐	‐	
Hispanic or Latino	1.2	0.8	1.8	0.43
Religion				
Catholic (referent)	‐	‐	‐	
Christian	0.4	0.3	0.7	<0.001
Employment				
Employed (referent)	‐	‐	‐	
Unemployed	3.0	1.9	4.8	<0.001
Retired	2.4	1.5	3.9	<0.001
Education level				
High school or GED (referent)	‐	‐	‐	
Did not complete high school	0.6	0.3	0.9	0.02
Some college, vocational or bachelor's	0.5	0.3	0.8	<0.01
Prefer not to answer	0.4	0.2	0.8	<0.01

On multivariable logistic regression of gender, age, education level and employment status, gender and education level were not significant predictors of preference. (Table 3). Similar to univariable analysis, age 25–40 was positively associated with gender preference while controlling for other variables. Being employed was associated with significantly less gender preference. Religion and race were not incorporated into the multivariable model, as 100% of Muslim patients indicated no provider preference, whereas 100% of White/Caucasian patients had a preference. The dependent variable of having a gender preference was used as a proxy for male provider preference, as only 18 patients (3%) had a female provider preference. All patients preferring a female provider were aged 41–60, female, Hispanic or Latino, Catholic, employed and completed a high school level of education.

**TABLE 3:** Multivariable logistic regression for patient preference

	OR	95% CI		*p* value
Gender (male)	1.5	0.7	3.2	0.27
Age (years)				
41–60 (referent)	‐	‐	‐	
25–40	25.7	10.4	63.6	<0.01
61–80	0.2	0.1	0.4	<0.01
>80	0.1	0.1	0.3	<0.01
Education level				
High school or GED (referent)	‐	‐	‐	
Did not complete high school	0.8	0.4	1.8	0.66
Some college or bachelor's	1.2	0.6	2.7	0.58
Employment status				
Unemployed (referent)	‐	‐	‐	
Employed	0.1	0.0	0.1	<0.01
Retired	1.7	1.0	3.0	0.056

## 4 DISCUSSION

Our study investigated patient factors that influence gender preference for urologic providers in a predominantly male (90%) cohort with high rates of Hispanic or Latino ethnicity (43%) and unemployment (40%). We found that current unemployment status and age 25–40 significantly predicted provider preference, with the majority of patients preferring a male urologist. Patient gender did not influence provider preference; female patients were no more likely to have a provider preference than men (OR 0.7, 95% CI 0.4–1.3, *p* = 0.29). Our study is not the first in the literature that surveyed urology patients on their preference for a male or a female provider. Amir et al.^9^ administered surveys to 119 male patients presenting to the clinic and collected basic sociodemographic information, gender preferences and the most important characteristics of their urologist. Patients exhibited more same‐gender preference for physical examination (38%) or urological surgery (35%) than for consultation (24%). Most patients (97%) preferred a same‐gender urologist because they felt less embarrassed. Of note, a prior exam by a female urologist was reported to correlate inversely to same‐gender preference from 48% to 26%, implying a positive experience. Although this study was limited by its homogeneous population, its conclusion that increasing exposure to female providers resulted in decreased prejudice towards female practitioners has also been seen in the nonurologic literature.^10^

When surveying more gender diverse populations, other studies have shown that female patients are more likely to have a gender preference. However, as was seen in our study, patients that have a gender preference are consistently more likely to prefer their own gender. For example, Tempest et al.^11^ performed one of the earliest surveys in 2005 of 429 patients (326 male and 103 female) presenting to a urology clinic in the United Kingdom. The majority (80%) of patients had no gender preference for their provider. Of those who did have a preference, 98% preferred the same gender, and female patients were more likely to have a gender preference than male patients (OR 1.8, 95% CI 1.07–3.03, *p* = 0.027). Another study from Korea performed a survey of 270 clinic patients and showed that women were more likely to have a gender preference than men (63.5% vs. 39.7%), with 62.3% of all female patients preferring a same‐gender urologist compared to 25.9% of all male patients. This preference was not correlated with age or education level.^12^ Siu explored barriers to communication between female patients and male urologists in Hong Kong and argued that female patients often felt misunderstood and embarrassed when being seen by male urologists, leading to a loss of autonomy in their care.^13^

A recent review found that overall there was a preference for a same‐gender urologic provider, with female patients being more likely to exhibit this preference. Much like the study by Siu et al., Tabatabai et al.^14^ found that female patients were inhibited by perceived insensitivity and embarrassment by male urologists. Oftentimes, this fear of the male provider caused female patients to delay care. Fear of being misunderstood and disconnect in communication are the most commonly cited reasons for this preference.^14^ This should be taken as a learning point for male and female urologists on the importance of empathy and open communication to ensure a healthy physician–patient relationship and appropriate care. Interestingly, a recent publication in JAMA Surgery^15^ showed that discordant gender between surgeon and patient resulted in adverse patient outcomes, with the most significant trend seen between male surgeons with female patients. Whereas no direct causative factors were explored in the study, the findings are concerning. As prior literature suggests, female patients have historically felt misunderstood by their male physicians, and this may translate into adverse surgical outcomes. Perhaps by understanding these socioeconomic determinants of patient preferences, physicians, especially surgeons, can provide more thoughtful care with better patient compliance as well as outcomes.

The 2020 American Urological Association (AUA)^16^ census demonstrated an increase in number of practising female urologists, growing to 1375 practitioners or 10.3% of the total workforce. This is the first time in history that female urologists surpassed 10% of all practising urologists. This increase in female urologists is seen as a result of more female graduates from urology residency and other training programmes. In fact, urology has seen the greatest increase in female representation from 1978 to 2013, with an 11‐fold increase, compared to other surgical specialties.^17^ A recent study by Nam et al.^18^ projected that the pool of female urologists will grow 3.77‐fold from 2020 to 2060, compared with the pool of male urologists which will grow 1.33‐fold. This increased presence of female urologists is commendable, as historically female practitioners have been found to have superior communication skills with patients and devote more time to psychosocial aspects of disease rather than only pathophysiological. Indeed, Gray et al.^19^ found that in the patient–doctor relationship, communication is easier, more time is given to patients, fewer drugs are prescribed and female patients are more likely to be taken seriously when the physician is a female. Whereas this study was not limited to relationships between patient and urologist, the findings are stimulating in that they suggest a pervasive positive culture of medical practice by female physicians that often gets neglected in the current medical literature.

The rise in visibility of female urologists makes studies like ours extremely prescient. By identifying biases patients may have for providers, we can work to dispel some myths to better equalise patient attitudes towards diverse physician genders. With an increasing number of female urologists, the preference of male patients for male urologists may be an obstacle for the rising generation of female urologists, if persistent.

Our study has many strengths. Our patient population is diverse, with multiple genders, ethnicities, religions and age groups represented. It is the largest survey of urologic clinic patients currently in the literature to explore what patient factors are most likely to influence preference for a male or a female provider. That being said, our study has its fair share of limitations. First, while we strove to achieve a heterogeneous population by including numerous disparate clinic sites and accrued over 500 patients, our response rate was skewed, with certain demographics preferentially choosing a gendered urologist in totality. This compromised our statistical analysis such that religion and race could not be used in multivariate analysis. Second, only 10% of respondents were female, which limits the amount of information we glean from this group. Specifically, our study was adequately powered only to detect a moderate‐sized effect (≥20% difference between genders). Therefore, if the true difference is small between male and female patients, we were underpowered for detecting this difference. Third, our respondents were largely underserved, living in a large, diverse, urban city, which may limit the generalisability of our findings and contribute to any differences in outcomes between our study and others. Finally, certain demographic or preference questions may be viewed as deeply personal, and as such, patients answering those questions may feel compelled to respond in a certain way out of concern for the reaction to their responses. This is an example of observer bias.

Understanding how patient sociodemographic factors influence provider preferences allows healthcare systems to provide more sensitive and culturally competent care. Our specific finding of increased preference for male providers in patients who had a lower education attainment (on univariate analysis) and were currently not employed (on univariate and multivariate analysis) should be closely examined at a state and national level. Government officials can use these findings to prioritise access to education and job opportunities, thereby potentially dispelling some of the misgivings patients might have towards female urologists. At the level of the urologic community at large, our results provide provocative evidence that better access to jobs and education may help normalise the disparity in patient preference for specific gender urologists. Urology residency programmes, academic institutions and hospital systems should bolster their female urologists and contribute further to the evolution of the field while being sensitive of these aforementioned patient demographic factors.

In summary, patient gender, race and religion do not appear to influence their preference to be seen by a male or a female urologist in the clinic setting. However, patient age, unemployment and potentially educational attainment were significantly associated with a provider gender preference. These findings suggest that expanding patient access to education and that increasing job opportunities are potential keys to dispelling the gender disparity in urologic provider preference. These initiatives in combination with the rising number of women in the physician work force will hopefully normalise the female presence in the field of urology and eliminate patient biases in provider gender preference.

## DISCLOSURE OF INTERESTS

None of the authors have any relevant disclosures, and none of the authors have any financial or non‐financial interests that may be relevant to the submitted work.

## Supporting information


**Supporting information S1**. Preference questionnaire.
