# Supplementary material for: Factors that influence preference for male or female urologist among underserved patients in New York City
Source: BJUI Compass. 2022 Oct 17;4(2):167–72. doi: 10.1002/bco2.196 (PMC9931541; doi:10.1002/bco2.196)
Supplement: Supplementary file 1 — Supporting information S1. Preference questionnaire. [file BCO2-4-167-s001.docx]

1. How old are you?:

- <25 years old
- 25-40 years old
- 41-60 years old
- 61-80 years old
- >80 years old
- Prefer not to answer

1. What gender do you identify with?

- Male
- Female
- Other (please specify): _________________________________________________

1. What is your race/ethnicity?

- American Indian/Alaskan Native
- Asian/Pacific Islander
- Black or African American
- Hispanic or Latino
- Native Hawaiian
- White/Caucasian
- Unknown/ Not reported
- Prefer not to answer
- Other (*please specify*): ________________________________________

1. Are you Hispanic or Latino?

- No
- Yes
- Don’t know
- Prefer not to answer

1. What is your religious affiliation, if any?

- Christian
- Catholic
- Jewish
- Hindu
- Muslim
- Buddhist
- Agnostic
- Atheist
- Prefer not to answer
- Other (please specify): __________________________

#### Which of the following best describes your current work status:

#### Employed (including self-employment)

#### Unemployed

#### Retired

#### Prefer not to answer

- Other *(please specify):* _______________

1. What is the highest level of education you attained?

- Less than 8 years (Grade 1-8)
- 8-11 years (Some high school without graduation)
- High school graduation or GED
- Vocational college or technical school
- Some college or university
- College degree (Bachelor’s degree)
- Graduate/Professional degree
- Prefer not to answer

1. What is the reason for your visit?

- Initial consultation
- Follow up visit
- Office procedure
- Surgical scheduling

1. Previously, have you been seen by a male or female urologic provider?

- Male
- Female
- Does not apply, this is first visit

1. How would you rate your experience with your prior provider? Select N/A if this is your initial consultation.

- Excellent
- Good
- Fair
- Poor
- N/A

1. Would you prefer to be seen today by a male or female urologic provider?

- Male
- Female
- No preference

1. If you were to have a physical exam today, would you prefer to have it performed by a male or female provider?

- Male
- Female
- No preference

1. If you were to have urologic surgery performed, would you prefer to have it performed by a male or female provider?

- Male
- Female
- No preference

1. If you were to have a clinic visit for consultation only, would you prefer to be seen by a male or female provider?

- Male
- Female
- No preference

1. Cuántos años tiene usted?:

- <25 años
- 25-40 años
- 41-60 años
- 61-80 años
- >80 años
- Prefiere no responder

1. Con qué género le identifica?

- Hombre
- Mujer
- Otro (por favor especifica): _________________________________________________

1. Cuál es su raza/etnia?

- India Americano/native de Alaska
- Asiático/isleño del Pacífico
- Negro o afroamericano
- Hispano o latino
- Nativo de Hawái
- Blanco/caucásico
- Desconocido/no informado
- Prefiere no responder
- Otro (*por favor especifica*): ________________________________________

1. Es Hispano o Latino?

- No
- Si
- No sé
- Prefiere no responder

1. Cuál es su afiliación religiosa, si la hubiera?

- Cristiano
- Católico
- Judío
- Hindú
- Musulmán
- Budista
- Agnóstico
- Ateo
- Prefiere no responder
- Otro (por favor especifica): ______________________________________________

1. Cuál de las siguientes opciones describe mejor su situación laboral actual

- Empleado (incluido el trabajo por cuenta propia)
- Desempleado
- Retirado
- Prefiero no responder
- Otro (*por favor especifica*): _______________

1. Cuál es el nivel más alto de educación que alcanzó

- Menos de 8 años (grados 1-8)
- 8-11 años (alguna escuela secundaria sin graduación)
- Graduación de la escuela secundaria o GED
- Colegio vocacional o escuela técnica
- Algún colegio o universidad
- Título universitario (licenciatura)
- Licenciatura / título profesional
- Prefiere no responder

1. Cuál es el motivo de su visita?

- Consulta inicial
- Visita de seguimiento
- Procedimiento de oficina
- Programación quirúrgica

1. Ha sido atendido anteriormente por un urólogo hombre o mujer?

- Hombre
- Mujer
- No pertanece, esta es la primera visita

1. Cómo calificaría su experiencia con su proveedor anterior? Seleccione N/A si esta es su consulta inicial.

- Excelente
- Bueno
- Promedio
- Mal
- N/A

1. Preferiría ser atendido hoy por un proveedor de urología masculino o femenino?

- Hombre
- Mujer
- Sin preferencia

1. Si tuviera que realizarse un examen físico hoy, ¿preferiría que lo realizara un proveedor masculino o femenino?

- Hombre
- Mujer
- Sin preferencia

1. Si le realizaran una cirugía urológica, ¿preferiría que la realizara un proveedor masculino o femenino?

- Hombre
- Mujer
- Sin preferencia

1. Si tuviera una visita a la clínica solo para consulta, ¿preferiría ser atendido por un proveedor masculino o femenino?

- Hombre
- Mujer
- Sin preferencia
